# Progress in Psychiatry

**Published:** 1903-02-14

**Authors:** 


*(To be continued.)*

### PROGRESS IN PSYCHIATRY.

**Heredity of Mental Disease in General.**—Dr. J. Wiglesworth, Medical Superintendent of Rainhill Asylum, deals at some length with important and practical facts collected in a long experience and referring to the question of the hereditary transmission of mental diseases.[1] It used to be taught in the earlier days of biological science that "the respective shares which the sperm-cell (spermatozoon) and the germ-cell (ovum) contributed to the act of fertilisation were of a different order, the germ-cell furnishing the matter, the sperm-cell supplying the force which animated that matter, and started it upon its career of development." This view can no longer be held in the light of modern research. We now know that the portion of the germ-cell or ovum which takes the main part in the formation of the fœtus or offspring is the nucleus. It is to the union of the nucleus of the sperm-cell with that of the germ-cell that the development of the fœtus is due, the two nuclei (male and female respectively) contributing equal shares, in the material sense, to the process. The nuclear material which thus constitutes the vehicle of transmission of hereditary qualities to the fœtus from both parents, is itself an organised and complex structure, into whose composition there enter probably many millions of molecules of multiform character, and which singly or in groups take part in the development of every portion of the fœtus. Prior to fertilisation the nucleus of the ovum undergoes a series of changes whereby one-half of the nuclear "chromatic substance" is divided off and extruded. Precisely analogous changes take place with regard to the nucleus of the spermatozoon with the exception that each half of the "chromatic substance" resulting from the division continues as a sperm-cell. Although the precise interpretation of these "reducing divisions" is still obscure, the net result is obviously to diminish by one-half the chromatic substance contained both in the germ-cell and in the sperm-cell. And it seems clear that the chief object of this process is to prevent indefinite increase in the mass of the nuclear substance, and thus to secure uniformity of mass. But this "reducing division" is not merely a process for keeping the mass of the nuclear substance uniform in size. For prior to the operation of "reducing division" the nuclear structures (chromatic rods) undergo a process of splitting or cleavage by which each chromatic rod is split longitudinally into halves. Weismann, whose monumental work[2] on heredity, has done much to illuminate the subject, believes that the object of this primary doubling of the nuclear rods is to increase the possible number of combinations of the germ-plasma, and therefore the number of varieties which result from the union of the parent cells after "reducing division" has been completed. This is clearly a factor of immense importance in phyletic development. Natural selection acts always and everywhere by seizing upon and fixing favourable varieties, and the greater the number of variations it has to work upon, the greater is the chance of a type being developed adapted to any particular environment. There can be no doubt that only a fraction of the countless variations which are being continually produced become permanently fixed, the rest being silently quenched by the operation of this great natural law. Hence the vast importance of obtaining the greatest possible diversity in the minute structure of the germ plasma, so as to furnish an abundant material for natural selection to work upon. The above observations will show that the problem of heredity is one of great complexity.

The problem of heredity is for the alienist one of particular interest and importance, for it daily demonstrated that mental (cerebral) peculiarities, no less than bodily ones, descend from parent to child, and that the aberrant mental traits of one generation are the logical sequence of the mental abnormalities of preceding generations. The practical questions which may be addressed to psychological medicine on this point are many. For example, what proportion of insane parents owe their insanity to definite hereditary taint? and in how many, on the other hand, can the insanity be said, in the strictest sense, to have been acquired in the course of the individual life? Is one sex more prone to hereditary insanity than the other? Is one parent more potent than the other in transmitting the disease to the offspring, and are the male and female children affected in different numerical proportions, according as their insanity is derived from the father or the mother? Do the different forms of insanity differ from one another in the degree with which they tend to be inherited? Can the result of disease acquired by a parent be transmitted to the offspring so as to appear in the latter in the form of a tendency to insanity? Is there any diathesis (other than insanity) occurring in one parent which, when associated with the insane diathesis in the other parent, might tend to neutralise transmission of the latter diathesis to the offspring, and, conversely, is there any diathesis which tends to reinforce the insane diathesis and to add, therefore, to the dangers? With the object of answering some of these questions an inquiry was made into the family history of a series of cases of insanity observed by Dr. Wiglesworth. These statistical inquiries deal with a series of 3,445 patients admitted to Rainhill Asylum during a period of 12 years, 1,693 of the patients being males, and 1,752 females. These were "patients concerning whose family history any details whatever were obtainable," and in whose direct parentage (fathers, mothers, and grandparents) or collaterals (brothers, sisters, uncles and aunts) insanity, if it was met with, was regarded as a hereditary factor. Patients whose cousins only afforded evidence of insanity were not included in the class of hereditary insanity, as it was thought that there was an equal chance of such taint having been introduced by marriage from an entirely different family, and it could not be assumed, without further proof, that such taint belonged to the family of the patient. "Out of the whole series of 3,445 patients a definite hereditary taint of insanity, epilepsy, or a marked degree of eccentricity or peculiarity, either direct or collateral, was found in 965 cases, a percentage on the whole number of 28·01." There can be no doubt that this figure errs, adds Dr. Wiglesworth, on the side of deficiency, as it is difficult to get trustworthy information as to the family history of our patients, especially when they belong to the pauper class. Farquharson,[3] a recent observer, dealing with a large number of patients of a similar class, obtained a percentage of hereditary taint of 30·7. Statistics drawn from institutions which receive private patients—as distinguished from paupers—usually give appreciably higher results. Thus Grainger Stewart[4] found a percentage of 49·6 among the patients admitted into the Crichton Royal Asylum. Reverting to Dr. Wiglesworth's figures, which give an hereditary taint of insanity in 28·01 per cent. or 965 out of 3,445 patients, it is found that the sexes exhibit a difference. Of these 965 patients, 419 were males and 546 females; and a simple calculation of these numbers in relation to the separate totals of male and female patients investigated in the report shows that the percentage of hereditary taint was 24·74 for male patients, and 31·16 for females. This striking difference has an interest which is enhanced by the fact that it harmonises with the records of previous investigators, almost all of whom have found a higher percentage of hereditary taint among the female patients than amongst the male. Thus Thurnam[5] gives a percentage of 32·82 for the males and 35·48 for the females; Grainger Stewart[6] gives 48·56 for males and 51·05 for females; while Farquharson[7] gives 27·4 per cent. for males and 34·16 per cent. for females. The conclusion has hence been drawn that the female sex has a greater liability to suffer from hereditary insanity than the male. It has been suggested that the female sex has to pass during life through physiological crises—puberty, pregnancy and the parturition, lactation, and the menopause—which constitute periods of danger, and which are prone to evoke into activity any latent hereditary taint of insanity, whereas in the male sex such danger is over, when the age of sexual maturity (25 years) is attained. It appears, however, that certain forms of insanity, to be mentioned later, are more often acquired in the lifetime of the individual in males than in females, and that when these are excluded the difference between the sexes as regards the hereditary incidence of insanity is not so marked.

[1] Jour. of Ment. Sci., Oct., 1902. [2] Contemp. Sci. Series, London, 1893. [3] Jour. of Ment. Sci. July, 1898. [4] Jour. of Ment. Sci., April, 1864. [5] Ibid. [6] Ibid. [7] Jour. of Ment. Sci., July, 1898.

